# Screening Methods for Isolation of Biocontrol Epiphytic Yeasts against *Penicillium digitatum* in Lemons

**DOI:** 10.3390/jof7030166

**Published:** 2021-02-25

**Authors:** Martina María Pereyra, Mariana Andrea Díaz, Fabricio Fabián Soliz-Santander, Anja Poehlein, Friedhelm Meinhardt, Rolf Daniel, Julián Rafael Dib

**Affiliations:** 1 Planta Piloto de Procesos Industriales Microbiológicos (PROIMI)—Consejo Nacional de Investigaciones Científicas y Técnicas (CONICET), Av. Belgrano y Pje. Caseros, 4000 Tucumán, Argentina; mmpereyra@conicet.gov.ar (M.M.P.); marianadiaz@conicet.gov.ar (M.A.D.); fabianfabriciosoliz@gmail.com (F.F.S.-S.); 2 Genomic and Applied Microbiology & Göttingen Genomics Laboratory, Institute of Microbiology and Genetics, Georg-August University of Göttingen, 37077 Göttingen, Germany; apoehle3@gwdg.de; 3 Institut für Molekulare Mikrobiologie und Biotechnologie (IMMB), Westfälische Wilhelms Universität Münster, 48149 Münster, Germany; meinhar@uni-muenster.de; 4 Facultad de Bioquímica, Instituto de Microbiología, Química y Farmacia, Universidad Nacional de Tucumán, Ayacucho 471, 4000 Tucumán, Argentina

**Keywords:** epiphytic yeasts, screening methods, biological control, *Penicillium digitatum*, citrus fruits

## Abstract

Worldwide, the green rot caused by *Penicillium digitatum* is one of the most aggressive postharvest diseases of lemons. Searching for sustainable alternatives to chemical fungicides, epiphytic yeasts as potential biocontrol agents were isolated from citrus fruits using a tailor-made selective medium. For disclosing their antagonistic potential against *P. digitatum*, obtained isolates were subjected to direct screening methods, both in vitro and in vivo. In the course of the primary in vitro screening that comprised dual culture assays, 43 yeast strains displaying antagonistic activities against the pathogen were selected. Subsequently, such strains were subjected to an in vivo screening that consisted of a microscale test, allowing the selection of six yeast strains for further analysis. In the final screening using macroscale in vivo tests, three strains (AcL2, AgL21, and AgL2) displaying the highest efficiencies to control *P. digitatum* were identified. The protection efficiencies in lemons were 80 (AcL2), 76.7 (AgL21), and 75% (AgL2). Based on sequence analysis of the PCR amplified D1/D2 domains of the 26S rRNA genes, they were identified as representatives of the species *Clavispora lusitaniae*. Interestingly, the strains exhibited a broad action spectrum among citrus fruits as they were also able to combat the green mold disease in grapefruit and two orange varieties. The direct screening methods applied in this study favored the recovery of efficient candidates for application as biological control agents to combat fungal infestations of citrus fruits.

## 1. Introduction

Argentina is one of the main lemon-fruit-producing and exporting countries, with the Tucumán province being the lemon-producing hub contributing 78% of the total national production [1]. Lemons are frequently exposed to several phytosanitary issues that cause losses of up to 12% of the fresh fruit [1]; the green mold disease caused by *Penicillium digitatum* is the most important postharvest fungal infestation [2]. Traditionally, chemical control by the use of fungicides has been employed to control postharvest decays [3,4,5]. However, due to the emergence of fungicide-resistant pathogens in citrus-production areas [3,6], the upcoming bans on the use of postharvest fungicides [7], and the public demand to reduce and avoid pesticides, are urgent needs to develop sustainable alternatives and safer technologies for controlling postharvest rots. Moreover, the mentioned drawbacks of fungicides have led markets to increase their demands to restrict chemical compounds, thereby promoting organic production of fruits and vegetables [8].

The use of naturally occurring epiphytic antagonists on fruit surfaces as biological control agents against postharvest pathogens is one of the most feasible alternatives to traditional fungicides. Selecting epiphytic antagonists from the environment where they are intended to be applied implies better adaptive advantages of the microorganisms [9,10] and greater public acceptance [11]. Biocontrol agents such as bacteria, yeasts, fungi, and viruses can control plant diseases with direct or indirect antagonistic effects [12]. In particular, yeasts have attracted considerable interest due to their diverse mode of action, enabling them to combat fungal rots in fruit. The mode of action include wound colonization, competition for space and nutrients, inhibition of spore germination, secretion of extracellular enzymes [13,14,15,16,17], development of biofilms, production of siderophores or volatile compounds [18,19,20], and the killer phenotype [21] as direct mechanisms, as well as the induction of resistance in the respective plants [22] as an indirect mechanism. Indeed, the successful use of yeasts to control postharvest diseases has been reported for diverse crops such as apples [23,24], pears [25,26], grapes [27,28], strawberries [29,30], peaches [31], and citrus [21,32,33].

A crucial step in the development of commercial products based on biological control agents is the screening and identification of suitable candidates. In our previous study, native yeasts for the postharvest control of *P. digitatum* were isolated from the surface of lemons and washing water from a local packinghouse [21]. As the latter sources showed to be adequate for isolation of efficient antagonistic yeasts, the current study aimed to increase the number of biocontrol candidates by using novel approaches for the isolation of naturally occurring epiphytic yeasts. The antagonistic activities of the biocontrol candidates were evaluated using different screening methods, in which the direct impact on the growth of the pathogen was measured. Furthermore, the biocontrol efficiency of the new isolates against green mold in lemons and other citrus fruits was evaluated.

## 2. Materials and Methods

### 2.1. Fruits

Lemons belonging to Eureka cultivars (*Citrus limon* (L.) *Burm*), sweet oranges variety Westin (*Citrus sinensis*), tangerine oranges (*Citrus x tangerine*), and grapefruits (*Citrus x paradisis*) were harvested from local fields in Tucumán province, Argentina. The selected cultivars had not received any preharvest treatment with synthetic pesticides. Healthy fruits were transported to the laboratory to be directly used or were stored at 8 °C for not more than 4 days. Selected fruits were free of any noticeable injury or signs of rot and were homogeneous in size, shape, and ripeness.

### 2.2. Pathogen

A phytopathogenic strain of *P. digitatum* belonging to the Phytopathology Lab of the citrus company San Miguel SA (Tucumán, Argentina) was used to generate green rot in lemons for the in vivo tests. The spore suspensions were prepared by collecting spores from a 10-day-old culture grown on PDA medium (4 gL^−1^ potato extract, 20 gL^−1^ glucose, 15 gL^−1^ agar, pH 5.6) at 25 °C. A total of 3 mL of saline solution containing 0.1% Tween 80 was added to the surface of the mycelium and scraped with a sterile loop. Spores were collected, and the suspension was adjusted to an OD_600_ of 0.1, which corresponds to a concentration of 10^6^ spores mL^−1^ [21].

### 2.3. Isolation of Epiphytic Yeasts

Isolation of yeasts with potential antagonism against *P. digitatum* was carried out following two different strategies. In the first one, a non-selective method similar to that described by Chalutz and Wilson [34] was used. Sources of possible antagonistic agents were sampled from two different stages of fruit processing from a lemon packinghouse: 100 mL of water from the first fruit washing station (FWS) and 100 mL of essential oil from the first essential oil extraction station (EOES). Both types of samples were serially diluted, and 100 μL of each dilution were subsequently plated in Petri dishes containing a modified YEPD-based medium called YEPD-CITRUS (5 gL^−1^ yeast extract, 10 gL^−1^ peptone, 20 gL^−1^ glucose, 20 gL^−1^ agar, 0.1% lemon essential oil, 1% lemon dehydrated peel powder, pH 4.5) to simulate the natural environment of epiphytic yeasts. It was also supplemented with ampicillin (100 μgmL^−1^) and chloramphenicol (50 μgmL^−1^) to avoid bacterial contamination. After 24–72 h of incubation at 25 °C, individual colonies with different morphological appearances were purified, examined under bright field microscopy, and stored in 20% glycerol at −80 °C. The second isolation protocol was based on a selective method similar to the one described by Wilson et al. [35]. Twenty freshly-harvested lemons were first sanitized with a 70% ethanol solution and wounded on the equatorial side (3 mm deep and 2 mm wide) using an awl. Fruit wounds were directly inoculated with a 20 μL sample (FWS or EOES). After 48 h incubation at 25 °C, 20 μL of the *P. digitatum* spore suspension was applied to each wound. Treated lemons were further incubated at 25 °C with high relative humidity (95%) for 5 days. Wounds without evidence of green mold were scraped and washed with sterile saline solution (0.85%). The obtained samples were serially diluted before spreading on YEPD-CITRUS plates and incubated at 25 °C for 24–72 h. Individual colonies were purified and stored as previously described.

### 2.4. Selection of Antagonistic Yeasts

#### 2.4.1. In Vitro Antagonistic Activity of Epiphytic Yeasts Against *P. digitatum*

Isolated epiphytic yeasts were screened for their ability to inhibit fungal growth on PDA plates using a slightly modified dual culture assay [36]. A total of 5 μL of the fungal spore suspension (10^6^ spores mL^−1^) was placed in the center of the Petri dishes (90 mm diameter). On the other hand, a loop of tested yeast was streaked as a strip 20 mm from the edge and 25 mm from the central drop. The negative control consisted of PDA plates inoculated only with the fungal spore suspension. Plates were incubated at 25 °C for 10 days, and the relative degree of mycelial growth inhibition was calculated according to the diameter measurement data, comparing the growth diameter of the fungus in the dual culture with the growth of the fungus in the control. Experiments were conducted in triplicates.

#### 2.4.2. Biocontrol Assay on Lemon Fruit Against *P. digitatum*: Microscale and Macroscale Tests

The efficiency of yeasts in wound protection against *P. digitatum* in lemons was first studied according to a microscale technique proposed by Ferraz et al. [37]. For this test, the best yeast candidates were selected based on the ability to inhibit *P. digitatum* in the in vitro tests. Yeast suspensions were prepared in 1 mL of saline solution using 24 h YEPD liquid cultures (10^8^ cells mL^−1^). Fifteen lemons per strain were surface disinfected, air-dried, and a single wound was introduced in the equatorial zone, as previously described. A total of 20 µL-aliquots of cell suspensions were inoculated into each wound, and treated fruits were incubated in a chamber under controlled conditions (25 °C for 24 h) before being inoculated with 20 µL of the *P. digitatum* spore suspension. Fruits were stored in covered plastic containers for 5 days with 95% relative humidity. The infection control consisted of 15 lemons treated only with the pathogen. After the incubation period, the protection efficiency of tested yeasts was evaluated according to the number of healthy lemons per treatment using the following equation:

Protection efficiency (%) = number of healthy fruit/total number of fruit(1)

The best yeast candidates from the microscale screening method were employed for a further macroscale assay [21]. In this case, cell cultures were grown for 24 h in liquid YEPD, and aliquots were transferred to Erlenmeyer flasks containing 250 mL of the same medium. They were incubated for 48 h with shaking. Afterwards, yeast cells were recovered by centrifugation at 8000× rpm for 5 min at 10 °C (SLA-1500 rotor, Sorvall Instruments RCSC, Du Pont, Wilmington, DE, USA) and resuspended in standard saline solution reaching a final concentration of 10^8^ cells mL^−1^. A total of 60 lemons per yeast (4 replicates of 15 lemons) were used. Fruits were disinfected and wounded as described above and then placed in net bags to be immersed in the yeast suspensions. Yeast-treated lemons were incubated in a controlled chamber for 24 h at 25 °C and, subsequently, immersed for 2 min in the fungus spore suspension. Yeast wound protection efficiency was evaluated after 5 days of incubation as detailed in the microscale test. A total of 20 lemons treated only with the pathogen served as the infection control in this assay.

Data were analyzed by ANOVA, and the mean values were compared with Tukey’s test at the 5% significance level. The InfoStat/L software (Córdoba, Argentina) [38] was used for the statistical analysis.

### 2.5. Yeast DNA Extraction

DNA extraction from isolated yeasts was performed following the methodology of Silverman [39] with slight modifications. Cell cultures were grown in 10 mL of YEPD medium at 25 °C under shaking (170 rpm) for 48 h, and 1 mL of each culture was pelleted by centrifugation at 13,000× rpm (SLA1500 rotor, Sorvall Instruments RCSC, Du Pont) for 5 min. Supernatants were discarded. The recovered pellets were incubated for 1 h at 37 °C in a solution containing 500 µL of Sorbitol (1 M), 100 µL of EDTA buffer (pH 7.5), and 10 µL of zymolyase (2.5 mgmL^−1^, Zymo Research, Irvine, CA, USA). After incubation, samples were centrifuged under the above-mentioned conditions. The resulting pellets were suspended in 500 µL of Tris-EDTA buffer (0.05 M Tris, 0.02 M EDTA, pH 7.4) and 50 µL of 10% SDS before incubation at 65 °C for 30 min. Subsequently, 200 µL of 5 M potassium acetate was added, followed by incubation on ice for 1 h and centrifugation as described above. DNA precipitation from supernatants was carried out by adding one volume of isopropanol at room temperature (5 min, 25 °C), followed by centrifugation (10 min, 13,000× rpm, SLA1500 rotor, Sorvall Instruments RCSC, Du Pont). Pellets were washed twice with 70% ethanol and allowed to dry. Finally, they were suspended in 100 μL of TE buffer solution (10 mM Tris, 1 mM EDTA, pH 7.4). DNA samples were analyzed using agarose (0.8% wv^−1^) gel electrophoresis and stored at −20 °C until further use.

Taxonomic identification was performed by PCR amplification of the D1/D2 domain of the 26S rRNA gene using primers NL-1 (5′-GCA TAT CAA TAA GCG GAG GAA AAG-3′) and NL-4 (5′-GGT CCG TGT TTC AAG ACG G-3′) [40]. The PCR amplification mix (final volume, 50 μL) contained: 50–100 μg μL^−1^ of purified genomic DNA, 0.5 μM of each primer, 200 μM of deoxyribonucleoside triphosphate (dNTPs), 1× of Phusion High Fidelity buffer, and 0.02 UμL^−1^ of Phusion DNA polymerase. The conditions to carry out the amplification were the following: initial denaturation at 98 °C for 30 s, 30 cycles of 10 s at 98 °C, annealing at 63 °C for 30 s, extension at 72 °C for 15 s, and the final extension at 72 °C for 5 min. Amplified products were analyzed by 1% (wv^−1^) agarose gel electrophoresis. Sequencing of the purified PCR products was performed at Microsynth Seqlab (Göttingen, Germany). The obtained sequences were processed using Clone Manager 9 Software (Cary, NC, USA), and sequence similarity searches were performed with the BLAST network service of the NCBI database (http://www.ncbi.nlm.nih.gov/BLAST). The sequences of these isolates have been deposited in the GenBank database under the following accession numbers: MT649495.1 (AcL2), MT649496.1 (AgL2), MT649498.1 (AgL21), MT649499.1 (AgRL4), MT649500.1 (AgRL5), and MT649497.1 (AgRL11).

### 2.6. Yeasts Protection Efficiency Against P. digitatum in other Citrus Fruits

The biocontrol spectrum of the selected yeasts against *P. digitatum* was evaluated using sweet oranges, tangerine oranges, and grapefruits. The experiment and the data analysis were carried out with the macroscale assay as described for lemons.

## 3. Results

### 3.1. Isolation and in Vitro Screening of Potential Biocontrol Epiphytic Yeasts

**Figure 1:**
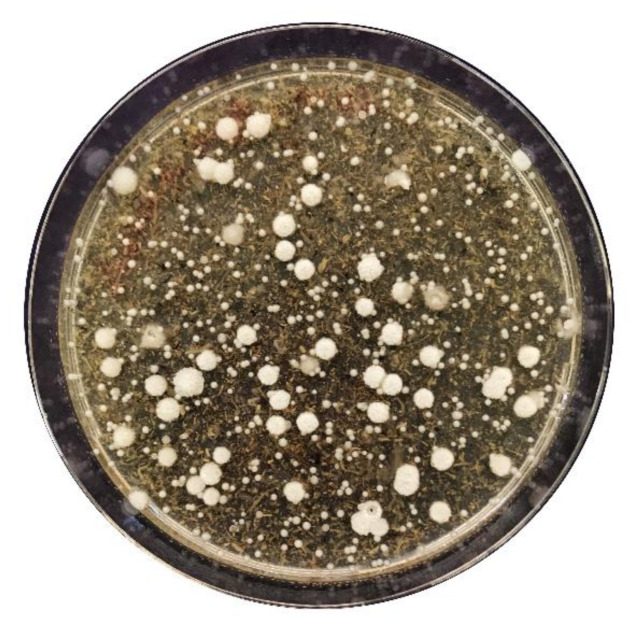
Yeast isolation on an agar plate using a modified YEPD medium. Yeast isolation from different antagonists’ sources was carried out in a modified YEPD medium added with lemon dehydrated peel powder and lemon essential oil (YEPD-CITRUS). Selection was based on different morphological appearances, and they were examined under bright-field microscopy to confirm yeast morphology.

Isolation of epiphytic yeasts was carried out using a novel modified YEPD medium added with lemon dehydrated peel powder and lemon essential oil (Figure 1). Two isolation strategies were adopted: a non-selective method in which the sources of antagonists were the FWS and the EOES; and a method whose selection was based on wounds of uninfected lemons, treated first with the foregoing samples and then with the pathogen. A total of 80 yeast strains were isolated: 56 from the non-selective method and 24 from the selective one (Table S1).

**Figure 2:**
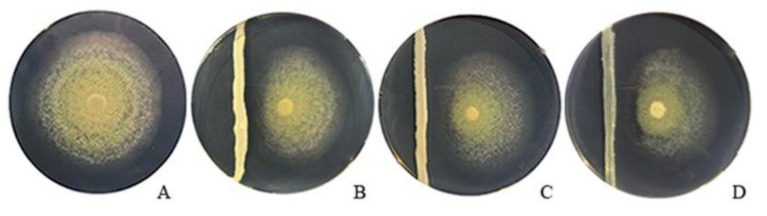
In vitro inhibitory activity of three isolated yeasts against *P. digitatum* on PDA medium after 10 days’ incubation at 25 °C. (**A**) Control plate inoculated only with the pathogen. (**B**–**D**) Plates inoculated with the pathogen and a strip of yeasts: AgL2, AgL21, and AcL2, respectively.

All isolated yeasts were primarily selected in an in vitro dual culture assay against *P. digitatum.* The antagonistic activity was determined by measuring the fungus relative growth inhibition after 10 days of incubation (Figure 2). Most of the strains (43) caused mycelial growth inhibition greater than 40%, 22 caused inhibition between 15 and 40%, 5 showed less than 15% inhibition, whereas the rest (10) did not affect the development of *P. digitatum* (Table S2).

### 3.2. In Vivo Screening Methods against P. digitatum in Lemons

**Figure 3:**
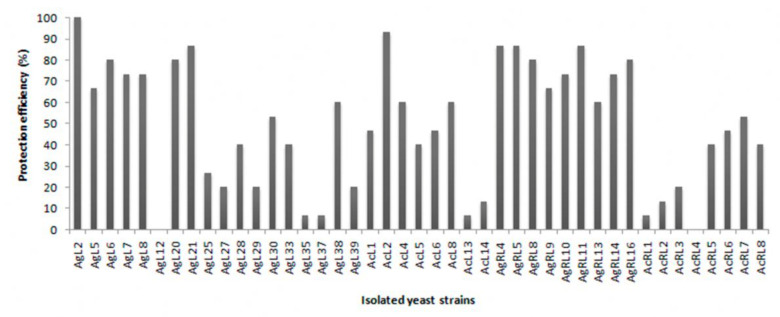
Wound protection efficiency of yeasts in the in vivo microscale assay. The 43 yeasts that most inhibited the mycelial growth of *P. digitatum* in the in vitro test were evaluated in a microscale assay against the pathogen after 5 days of incubation at 25 °C.

**Figure 4:**
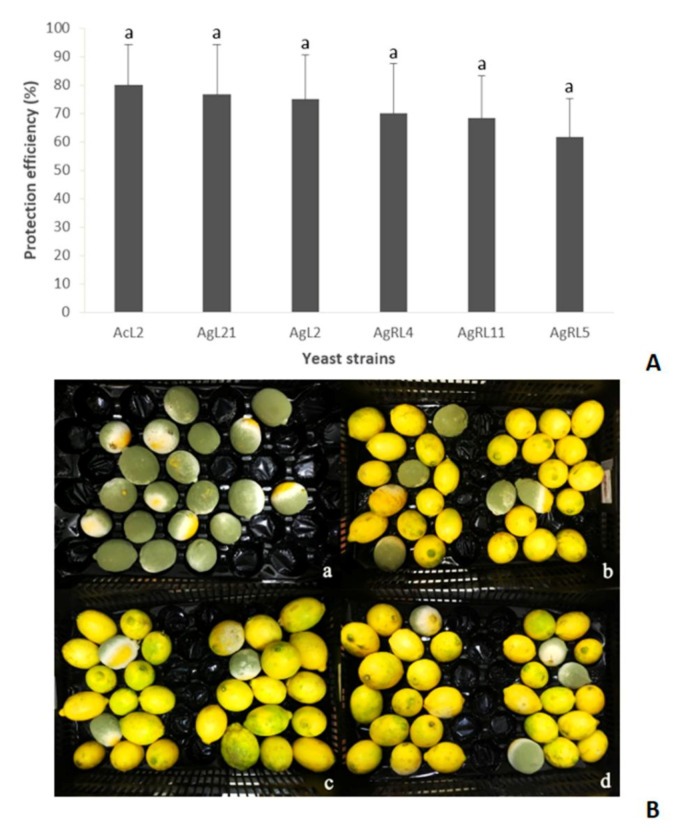
Wound protection efficiencies and biocontrol test of selected candidates. The upper panel (**A**) shows yeast wound protection efficiencies in the in vivo macroscale assay. The best yeast candidates obtained from the microscale assay were evaluated against *P. digitatum* after 5 days of incubation at 25 °C. Error bars indicate standard deviations. The bottom panel (**B**) represents the in vivo macroscale biocontrol test in lemons. The figure shows wound protection in lemons inoculated only with *P. digitatum* (**a**), compared to lemons pretreated with yeasts (**b**) AgL21, (**c**) AgL2, and (**d**) AcL2 after 5 days at 25 °C.

In vivo tests were conducted with the preselected isolates from the in vitro assay. The best 43 candidates were first evaluated in a microscale test against the pathogen. After 5 days of incubation at 25 °C, most of the yeasts were able to control green mold in wounded fruits (Figure 3). Wound protection efficiencies of at least 80% were adopted as the selection criterion for antagonistic yeasts to be further evaluated in the macroscale test, confirming their biocontrol activity against *P. digitatum*. Isolates that complied with this microscale test requirement were AgL2, AgL21, AcL2, AgRL4, AgRL5, and AgRL11. AcL2, AgL21, and AgL2 showed to be the most protective candidates in the in vivo macroscale test with efficiencies of 80, 76.7, and 75%, respectively (Figure 4).

### 3.3. Identification of Antagonistic Yeasts

According to the sequence analysis of the D1/D2 domain of the 26S rRNA gene and the search for similarities in the GenBank database, isolated yeasts were all affiliated to the Saccharomycetaceae family of the order Saccharomycetales. AgL2, AgL21, AcL2, AgRL4, AgRL5, and AgRL11 were identified as *Clavispora lusitaniae,* showing an identity greater than 99.5% to the reference strain *C. lusitaniae* Y8 (MN648842.1) (Table 1).

### 3.4. Biocontrol Efficiency against P. digitatum in other Citrus Fruits

**Figure 5:**
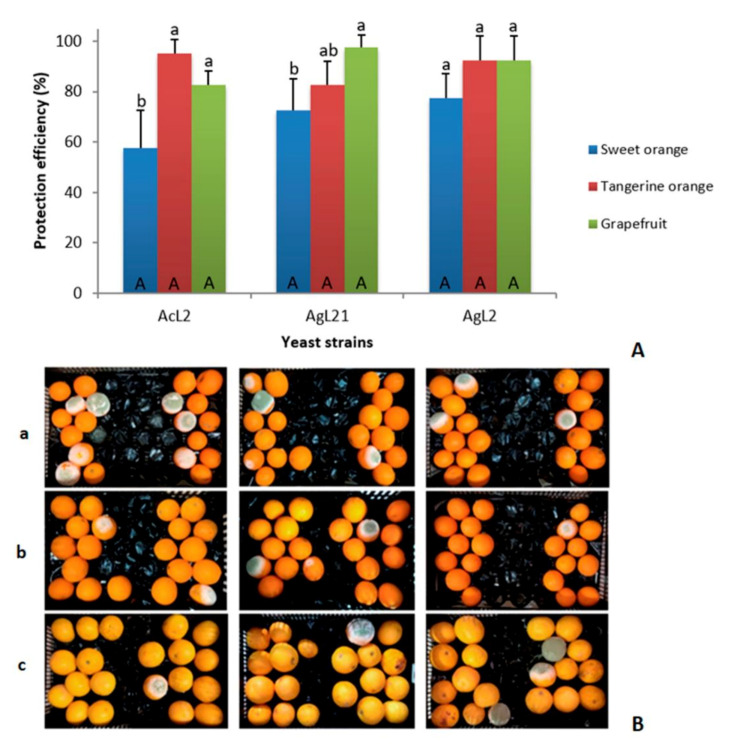
Protection efficiencies of potential yeast candidates against *P. digitatum* in citrus fruits. The upper panel (**A**) shows the protection efficiency of the three isolates against the pathogen in citrus cultivars. Mean values marked with identical letters are, according to the Tukey test (*p* < 0.05), not significantly different. Lowercase letters compare the efficiency of each yeast in the three citrus varieties. Uppercase letters represent the efficiencies comparison among the three yeasts in the same variety. The bottom panel (**B**) represents the in vivo test after 5 days of incubation at 25 °C. The efficiency of the yeasts AcL2, AgL21, and AgL2 (sorted by column, respectively) was evaluated in sweet oranges (**a**), tangerine oranges (**b**), and grapefruits (**c**).

The three candidates selected for the in vivo macroscale assay were evaluated with respect to protection activity against green mold in other citrus fruits to assess their biocontrol spectrum. Yeasts showed efficiency in controlling green mold in sweet oranges, tangerine oranges, and grapefruits (Figure 5). AcL2 and AgL21 showed the highest protection efficiency for tangerine oranges and grapefruits (95 and 97.5%, respectively), whereas sweet oranges protection was significantly lower in both cases (AcL2 57.5% and AgL21 72.5%). Strain AgL2 was able to control the green mold in all tested citrus, with protection efficiencies of 77.5, 92.5, and 92.5% for sweet oranges, tangerine oranges, and grapefruits, respectively. It should be noted that there were no significant differences in protection when comparing the protective effects of AcL2, AgL2, and AgL21 against the same type of citrus fruit.

## 4. Discussion

The drawbacks associated with the use of synthetic fungicides in the control of postharvest fungal diseases in lemons, followed by the growing demand for organic products, have encouraged the search and development of effective and more sustainable alternatives for the control of postharvest decays. In this regard, biological control agents based on yeasts have shown great potential as an alternative to the use of fungicides.

The main objective of this study was to isolate and select potential biocontrol yeasts to prevent or reduce infection by *P. digitatum* by using different direct screening methods. Most studies report the isolation of biological control agents from the region in which the final application is intended. This strategy is recommended to obtain microorganisms adapted to the environment, ensure their survival, and enhance their biocontrol activity in terms of their possible commercialization [11,41]. The isolation strategies described in this study allowed the selection of efficient antagonistic yeasts for the control of postharvest diseases of fruits. On the one hand, the presence of candidate yeasts was determined by a non-selective method in which the source of antagonistic agents was directly the FWS and the EOES. On the other hand, a selective isolation method was carried out from the same samples. Our results showed that the highest number of isolates was obtained from the non-selective method (Table S1). Of the yeasts isolated by the selective method, 70.8% had antagonistic activity against *P. digitatum*, whereas only 46.4% of those obtained by the non-selective approach showed such activity (Table S2). These results are in line with those of Wilson et al. [35] and Huang et al. [42], who argued that the selective isolation method is highly effective and should be considered as a first option for the sampling of biocontrol agents. Similarly, Taqarort et al. [43] obtained a high number of antagonistic yeasts using a selective method. Additionally, a modified medium was used to promote yeast development by adding lemon peel powder and lemon essential oil. This enrichment strategy proved to be an efficient approach to isolate yeasts that could serve as potential biocontrol agents against postharvest diseases of lemons. Similarly, Vero et al. [23] isolated yeasts capable of colonizing apple wounds and prevent the development of *Penicillium expansum* and *Botrytis cinerea* by adding apple juice to the culture medium.

Regarding the initial screening method used, the primary selection of biological control agents through in vitro dual culture assays has proven to be a simple, fast, and reproducible way to identify microorganisms with confirmed in vivo biocontrol activity [21,23,43]. In this study, 43 isolated yeasts were able to inhibit more than 40% of the mycelial growth of *P. digitatum* in the in vitro screening. However, it is important to mention that the screening of biocontrol agents by a dual culture assay is obviously restricted to those with direct activity against the pathogen, limiting the possibility of finding other promising biocontrol agents [41].

In a second selection screening consisting of a microscale in vivo test, in which both yeast and fungus were applied directly onto the lemon wound, the number of possible yeast candidates against *P. digitatum* was considerably reduced. The best yeast strains were selected according to their highest wound protection efficiencies in the microscale test: AgL2 100%, AcL2 93.33%, AgL21, AgRL4, AgRL5, and AgRL11 86.67%. Several studies reported the finding of successful antagonistic yeasts by employing such a methodology for controlling *B. cinerea* in apples [15] and grapes [44], *P. digitatum* in citrus [45,46,47], and *P. expansum* in pears [25]. Yeast strains AgL2, AcL2, AgL21, AgRL4, AgRL5, and AgRL11 not only inhibited the mycelial growth of *P. digitatum* in vitro but also prevented the pathogen development in fruits. Nevertheless, it should be noted that when the same yeasts were tested in the macroscale in vivo assay, their biocontrol efficiencies were lower, reaching values ranging from 61 to 80%. This highlights the importance of both choosing a suitable screening method for selecting yeasts and correctly selecting how to apply the biocontrol agent. For example, inoculation of the microbial agent directly into the fruit wound by the microscale method is a widely used technique due to its speed and easiness, but it certainly does not represent conventional application methods used under fruit packaging conditions.

In this study, the most promising yeast isolates (AgL2, AcL21, AgL21, AgRL4, AgRL5, and AgRL11) were identified by sequencing the D1/D2 region of the 26S rRNA gene and were all identified as representatives of *Clavispora lusitaniae*. This is consistent with our previous reports [21] in which *C. lusitaniae* strain 146 acted as an efficient biological control agent against *P. digitatum*, including fungicide-resistant *P. digitatum* strains [48]. In the present study, new methodologies have been tested to favor the isolation and selection of biocontrol agents: (i) the isolation of yeasts from two different samples (the FWS and the EOES), (ii) the use of a modified culture medium to simulate the natural environment of lemon epiphytic yeasts, favoring their development, (iii) the use of a selection method to obtain yeasts from uninfected lemon wounds previously inoculated with the industrial samples described here, and (iv) the implementation of different direct screening methods for an appropriate selection of candidate yeasts as biocontrol agents. Hence, these novel procedures led us to isolate members of the most efficient genus according to our own previous studies [48,49]. Apparently, members of the species *C. lusitaniae* seem to be native citrus yeasts with strong protective impacts on green rot in lemons. Furthermore, our group demonstrated recently that *C. lusitaniae* 146 is highly tolerant to certain stress factors associated with lemon storage and packaging processes, such as oxidative stress, fruit drying temperature, salts, and disinfectants commonly used in the citrus industry as well as UV-B irradiation [50]. Resistance to various stressors could explain the abundance of these yeast species in the isolation sources.

*C. lusitaniae* strains AgL2, AgL21, and AcL2 behaved as broad control agents among citrus fruits, as other than lemons, they controlled the green mold in oranges and grapefruits as well. The selection of a biocontrol agent with a broad spectrum of activity is a commercially highly desirable trait [51], which increases the application possibilities of formulations based on such yeasts. It is, thus, not surprising that other biocontrol yeasts such as *Aureobasidium pullulans* [52] and *Candida oleophila* [53,54], available in commercial formulations [17], also exhibit a wide spectrum of action in different crops.

## 5. Conclusions

By employing novel isolation and screening approaches, it was possible to obtain native epiphytic yeasts with effective antagonistic activity from citrus sources. It is noteworthy that any selection method is driven by certain interests and, hence, will be selective, which implies that not all candidate microorganisms suitable to act as biocontrol agents will be detected. The *C. lusitaniae* isolates AcL2, AgL2, and AgL21 were the most efficient in controlling the most important postharvest pathogen of lemons, both in vitro and in vivo. In addition, they proved to be agents with a broad activity spectrum, managing to control green mold in different varieties of citrus. Thus, the selected strains expand the collection of candidate yeasts for possible applications as alternative biological control agents against postharvest fungal diseases. Currently, in addition to evaluating the safety of the biocontrol yeasts with respect to human health, the mechanisms of action by which these new isolates exert their biocontrol activity are being studied, which certainly will also contribute to the understanding of the protective effects of yeasts against other postharvest citrus phytopathogens.

## Figures and Tables

**Table 1:** Identification of the yeast species of the six best isolates.

Isolate	Fragment Length ^a^	Species Designation	GenBank Accession Number	Identity (%) ^b^
AcL2	461	*Clavispora lusitaniae*	MT649495.1	100
AgL2	461	*Clavispora lusitaniae*	MT649496.1	100
AgL21	461	*Clavispora lusitaniae*	MT649498.1	100
AgRL4	459	*Clavispora lusitaniae*	MT649499.1	99.57
AgRL5	461	*Clavispora lusitaniae*	MT649500.1	99.57
AgRL11	459	*Clavispora lusitaniae*	MT649497.1	99.57

^a^ Values refer to the number of base pairs per fragment; ^b^ Identical nucleotides percentage in the sequence obtained from the D1/D2 region of the 26S rDNA gene and the sequence found in GenBank.

## Data Availability

The data presented in this study are available in insert article or supplementary material here.

## Supplementary Materials

The following is available online at https://www.mdpi.com/2309-608X/7/3/166/s1, Table S1: Number of isolated yeasts according to source and employed method; Table S2: Relative growth inhibition of yeast strains against *P. digitatum*.
