# Supplementary material for: Screening Methods for Isolation of Biocontrol Epiphytic Yeasts against Penicillium digitatum in Lemons
Source: J Fungi (Basel). 2021 Feb 25;7(3):166. doi: 10.3390/jof7030166 (PMC7996618; doi:10.3390/jof7030166)
Supplement: Supplementary file 1 [file jof-07-00166-s001.pdf]

Table S1. Number of isolated yeasts according to source and employed method

Method	Source	No. isolated strains	Total
Non-selective	FWS	40	56
	EOES	16	
Selective	FWS	16	24
	EOES	8	

Table S2. Relative growth inhibition of yeast strains against *P. digitatum*

Strain	Relative growth inhibition	Isolated method: source
AgL1	0	
AgL2	+++	
AgL3	++	
AgL4	+	
AgL5	+++	
AgL6	+++	
AgL7	+++	
AgL8	+++	
AgL9	++	
AgL10	++	
AgL11	++	
AgL12	+++	
AgL13	++	Non-selective method: FWS
AgL14	0	
AgL15	++	
AgL16	0	
AgL17	++	
AgL18	++	
AgL19	++	
AgL20	+++	
AgL21	+++	
AgL22	++	
AgL23	++	
AgL24	0	
AgL25	+++	

AgL26	+	
AgL27	+++	
AgL28	+++	
AgL29	+++	
AgL30	+++	
AgL31	0	
AgL32	++	
AgL33	+++	
AgL34	++	
AgL35	+++	
AgL36	+	
AgL37	+++	
AgL38	+++	
AgL39	+++	
AgL40	0	
AcL1	+++	Non-selective method: EOES
AcL2	+++	
AcL3	++	
AcL4	+++	
AcL5	+++	
AcL6	+++	
AcL7	++	
AcL8	+++	
AcL9	0	
AcL10	++	
AcL11	0	

AcL12	++	
AcL13	+++	
AcL14	+++	
AcL15	+	
AcL16	+	
AgRL1	0	
AgRL2	++	
AgRL3	++	
AgRL4	+++	
AgRL5	+++	
AgRL6	++	
AgRL7	0	
AgRL8	+++	Selective method:
AgRL9	+++	FWS
AgRL10	+++	
AgRL11	+++	
AgRL12	++	
AgRL13	+++	
AgRL14	+++	
AgRL15	++	
AgRL16	+++	
AcRL1	+++	
AcRL2	+++	
AcRL3	+++	Selective method:
AcRL4	+++	EOES
AcRL5	+++	
AcRL6	+++	

AcRL7	+++
AcRL8	+++

+++: Growth inhibition > 40%; ++: growth inhibition between 15 and 40%; +: growth inhibition < 15%; 0: no growth inhibition.
